# Gene rearrangements in hormone receptor negative breast cancers revealed by mate pair sequencing

**DOI:** 10.1186/1471-2164-14-165

**Published:** 2013-03-12

**Authors:** Xiang Jiao, Sean D Hooper, Tatjana Djureinovic, Chatarina Larsson, Fredrik Wärnberg, Christian Tellgren-Roth, Johan Botling, Tobias Sjöblom

**Affiliations:** 1 Department of Immunology, Genetics, and Pathology, Uppsala University, Uppsala, SE 751 85, Sweden; 2 Current address: The Breakthrough Breast Cancer Research Centre, The Institute of Cancer Research, 237 Fulham Road, London, SW3 6JB, UK; 3 Department of Surgical Sciences, Uppsala University, Uppsala, SE 751 85, Sweden

## Abstract

**Background:**

Chromosomal rearrangements in the form of deletions, insertions, inversions and translocations are frequently observed in breast cancer genomes, and a subset of these rearrangements may play a crucial role in tumorigenesis. To identify novel somatic chromosomal rearrangements, we determined the genome structures of 15 hormone-receptor negative breast tumors by long-insert mate pair massively parallel sequencing.

**Results:**

We identified and validated 40 somatic structural alterations, including the recurring fusion between genes *DDX10* and *SKA3* and translocations involving the *EPHA5* gene. Other rearrangements were found to affect genes in pathways involved in epigenetic regulation, mitosis and signal transduction, underscoring their potential role in breast tumorigenesis. RNA interference-mediated suppression of five candidate genes (*DDX10*, *SKA3*, *EPHA5*, *CLTC* and *TNIK*) led to inhibition of breast cancer cell growth. Moreover, downregulation of *DDX10* in breast cancer cells lead to an increased frequency of apoptotic nuclear morphology.

**Conclusions:**

Using whole genome mate pair sequencing and RNA interference assays, we have discovered a number of novel gene rearrangements in breast cancer genomes and identified *DDX10*, *SKA3*, *EPHA5*, *CLTC* and *TNIK* as potential cancer genes with impact on the growth and proliferation of breast cancer cells.

## Background

The progression from normal cell, subject to stringent growth controls, to an unregulated tumor cell is a stepwise process of accumulating mutations and rearrangements in the genome [1], which may disrupt, inhibit or deregulate genes, or create novel fusion genes. However, to distinguish genetic alterations which confer strong advantages to the tumor cell (drivers) from the more numerous neutral aberrations (passengers) is a difficult task. Next-generation sequencing has revealed somatic mutations that may contribute to breast tumorigenesis [2-10]. Several whole genome sequencing studies aiming at detection of chromosomal alterations in cancer genomes have been carried out in the past few years thanks to the advances in massively parallel sequencing technology [2,4,6,7,11,12]. The widely adopted method for clinical classification of breast cancer subtypes is usually based on immunohistochemical (IHC) analysis of estrogen receptor (ER), progesterone receptor (PR) and human epidermal receptor 2 (HER2), categorizing breast carcinomas into hormone receptor positive and HER2 negative (ER + and/or PR + and HER2-), hormone receptor positive and HER2+ (ER + and/or PR + and HER2+), hormone receptor negative and HER2 positive (ER-/PR-/HER2+) and triple negative breast cancer (TNBC, ER-/PR-/HER2-) subtypes. IHC classification is of great value in clinical practice to predict disease outcome as well as assign suitable targeted treatments to patients. Hormone receptor negative breast cancers, composed by HR-/HER2+ and TNBC subtypes, often correlate with poor prognosis [13]. HER2+ cases often respond well to treatment with trastuzumab, which is an inhibitor of HER2-dependent signaling. However, there is no efficient targeted therapy for TNBCs [3,5,6,12,14]. There are at least five molecular subtypes of breast cancers defined by gene expression profiles: luminal A, luminal B, HER2-enriched, basal-like and normal-like. HR + tumors are frequently of luminal A or luminal B subtypes, while receptor negative tumors are most frequently of HER2-enriched or basal-like subtypes [13,15]. Recent large-scale breast cancer genome studies have revealed that each molecular subtype has specific pattern of genomic alterations and notably, tumors of HER2-enriched and basal-like subtypes harbor many more rearrangements than the luminal A subtype, which primarily consists of receptor positive breast tumors [2].

In this work, we performed whole-genome sequencing on 15 hormone receptor negative breast cancers (Additional file 1) to detect somatic gene rearrangements. Long-insert mate pair sequencing with ~2.5 kb insert size was chosen for increased detectability. PCR and Sanger sequencing confirmation of selected structural variants identified 40 novel somatic gene rearrangements and 29 genes directly affected by these alterations. We also demonstrate the potential biological functions of some affected genes by these rearrangements by RNA interference (RNAi) in breast cell lines.

## Results

### Landscapes of rearrangement

Thirteen breast cancers were sequenced with Life Technologies SOLiD 3, from which a total of 119 Mb mate pair reads were obtained, corresponding to an average nucleotide coverage of ~0.3-fold and an average clone coverage of 8-fold per sample. Two additional breast tumors were sequenced with SOLiD 4 to a read depth of 3-fold nucleotide coverage and 80-fold clone coverage on average.

**Figure 1:**
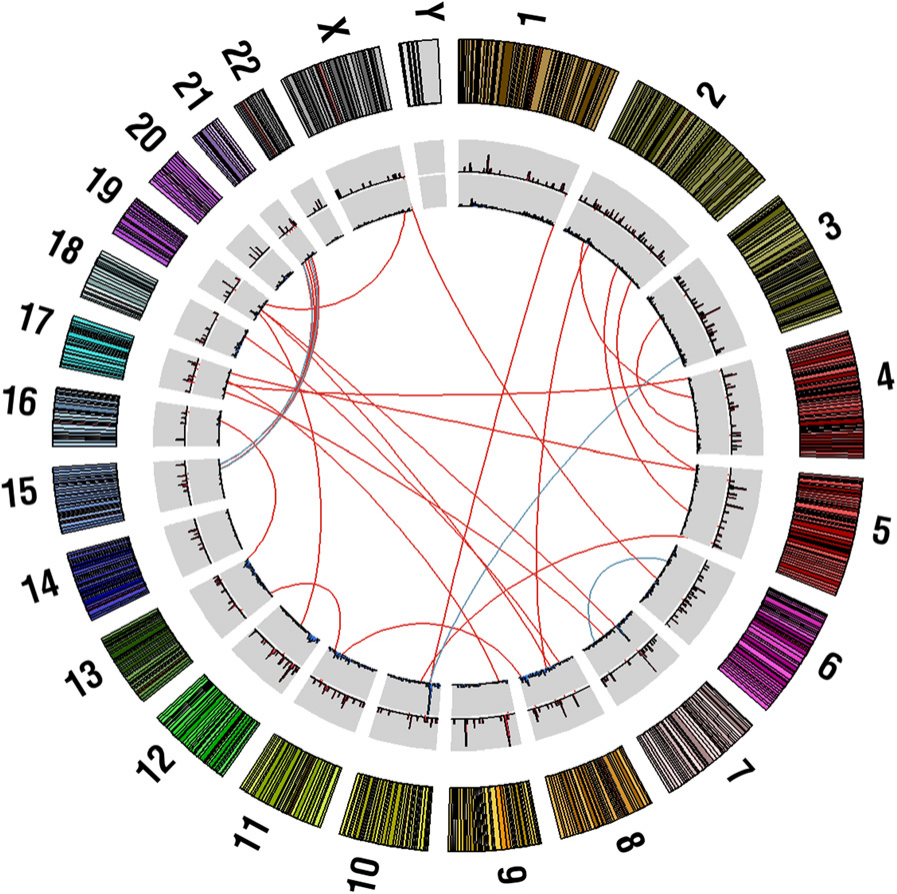
**Validated somatic rearrangements in breast cancer genomes.** Outer histogram of the Circos plot shows the number of deletions in each bin, and the inner histogram shows predicted insertions. Connections represent PCR validated (blue lines) and sequence validated (red lines) somatic translocations.

Structural variations (SVs) in the form of deletions, insertion, chromosomal translocations or inversions were observed in 8% of all mate pairs (range 3% - 15%, Additional file 2 and Additional file 3). The proportions and types of SVs vary among tumors, with two tumors having thousands of insertions (samples 120 T and 150 T, 3265 and 2466 insertions, respectively) while the other samples have much fewer, ranging from 3 to 260. In total, 165 putative rearrangements were selected for validation (Table 1), and 100 (61%) yielded products consistent with the predictions from the mate pair sequencing. Of these, 60 were also found in patient-matched normal tissue suggesting the presence of constitutional SVs, while 40 were observed only in tumor tissue and considered to be true somatic rearrangements (Figure 1, Table 2). Somatic SVs in individual tumors, including 8 deletions, 6 inversions and 26 interchromosomal translocations, are shown in Additional file 4. Interestingly, in one tumor sample (sample ID 118 T), we observed at least 5 validated translocations between chromosome 15 and 21, which may imply chromothripsis [16].

**Table 1:** Validation summary of deletions, inversions and translocations for each sample

**Tumor sample**		**Deletion**				**Inversion**				**Translocation**			
	**Attempted**	**Non-validated**	**Constitutional**	**Somatic**	**Attempted**	**Non-validated**	**Constitutional**	**Somatic**	**Attempted**	**Non-validated**	**Constitutional**	**Somatic**	
113 T	8	8	0	0	0	0	0	0	2	2	0	0
114 T	5	4	1	0	0	0	0	0	5	2	3	0
116 T	1	0	1	0	4	2	0	2	1	0	0	1
117 T	0	0	0	0	0	0	0	0	1	0	1	0
118 T	3	1	2	0	0	0	0	0	12	5	1	6
119 T	1	0	0	1	3	3	0	0	3	0	0	3
120 T	3	0	3	0	3	2	0	1	3	0	0	3
147 T	2	0	2	0	0	0	0	0	0	0	0	0
148 T	4	0	4	0	5	5	0	0	8	3	3	2
149 T	16	4	8	4	1	0	0	1	9	4	1	4
150 T	5	1	4	0	1	1	0	0	0	0	0	0
151 T	4	0	4	0	1	0	0	1	1	1	0	0
152 T	1	0	0	1	0	0	0	0	0	0	0	0
153 T	19	9	9	1	6	3	3	0	12	3	3	6
154 T	7	1	5	1	1	0	0	1	4	1	2	1

Due to the size of mate-pair inserts, insertions could not be validated by PCR. Attempted denotes the number of rearrangements attempted to validate, and Constitutional and Somatic denote the number of validated rearrangements in the normal and tumor samples validated by PCR followed by Sanger sequencing.

**Table 2:** Genes disrupted by sequence validated somatic rearrangements discovered in breast cancers

	**Sample ID**	**Chr 1**	**Breakpoint 1**	**Chr 2**	**Breakpoint 2**	**Disrupted genes**
						**(affected regions/breakpoint locations)**
Deletion	153 T	11	106618805		106622567	*GUCY1A2* (exon 7)
	154 T	8	72214170		72217978	*EYA1* (intron 7–8)
	119 T	5	134715922		134720809	*H2AFY* (intron 2–3)
	149 T	2	12526265		12528008	
	149 T	2	65366656		65369602	
	149 T	10	22889925		22892366	*PIP4K2A* (intron 3–4)
	149 T	15	45915722		45917969	
	152 T	12	2130488		2133385	
Inversion	120 T	2	120331908		120335057	*PCDP1* (intron 6–7)
	116 T	5	129549038		129552347	
	149 T	10	24436405		24438757	*KIAA1217* (intron 2–3)
	153 T	2	172893909		175831452	*METAP1D* (intron 1–2), *CHN1* (intron 1–2)
	116 T	3	168893755		170864487	*MECOM* (intron 2–3), *TNIK* (intron 12–13)
	151 T	5	137738030		137744577	*KDM3B* (intron 11–12)
Translocation	153 T	2	42052398	4	66411362	*EPHA5* (intron 3–4)
	153 T	6	104501617	X	152223450	
	153 T	7	54909974	19	29832131	
	153 T	8	57916723	19	30951050	*ZNF536* (intron 2–3)
	153 T	12	48517177	19	30945107	*PFKM* (intron 3–4), *ZNF536* (intron 2–3)
	153 T	19	30355201	X	153152399	*LCA10* (intron 5–6)
	153 T	5	174245601	10	65204015	*JMJD1C* (intron 1–2)
	116 T	2	199768975	5	28258969	
	118 T	7	90081994	17	25904801	*KSR1* (intron 3–4)
	118 T	15	60231305	21	47148999	*PCBP3* (intron 1–2)
	118 T	15	60833617	21	35917066	*RCAN1* (intron 1–2), *RORA* (intron 2–3)
	118 T	15	61356378	21	41870300	*DSCAM* (intron 3–4), *RORA* (intron 1–2)
	118 T	15	61375512	21	27982153	*RORA* (intron 1–2)
	118 T	15	71750638	21	18023846	*THSD4* (intron 6–7)
	119 T	2	42052196	4	66411644	*EPHA5* (intron 3–4)
	119 T	4	4714578	17	38990874	*TMEM99* (exon 3)
	119 T	8	32833459	18	64563574	
	120 T	8	127068558	11	112588800	
	120 T	11	108583473	13	21735983	*DDX10* (intron 10–11), *SKA3* (exon 5)
	120 T	13	103233303	16	77586406	
	148 T	2	65563071	8	80378299	*SPRED2* (intron 2–3)
	148 T	9	13526912	17	57745082	*CLTC* (intron 13–14)
	149 T	1	247997045	10	27944748	
	149 T	3	47806974	4	151298580	*LRBA* (intron 48–49), *SMARCC1* (intron 2–3)
	149 T	5	15946143	17	67928932	
	149 T	11	108583658	13	21742368	*DDX10* (intron 10–11), *SKA3* (exon 4)

Chr 1, the lower numbered chromosome in a rearrangement; Breakpoint 1, the breakpoint position on Chr 1 determined by Sanger sequencing; Chr 2, the other chromosome in a rearrangement; Breakpoint 2, the breakpoint position on Chr 2. Chromosome coordinates are based on human genome build HG19.

### Genes affected by rearrangements

Twenty-nine genes were predicted to be directly affected by the 40 validated somatic rearrangements, including genes previously reported to be altered in cancer as well as genes that have not yet been related to cancer (Table 2). Using Gene Ontology (GO) [17] as a reference for potential gene functions (Additional file 5), we discovered that these 29 affected genes are involved in multiple biological processes including epigenetic regulation (e.g. GO:0016568 chromatin modification), cell mitosis (e.g. GO:0007067 mitosis), signal transduction (e.g. GO:0007265 Ras protein signal transduction) and others.

**Figure 2:**
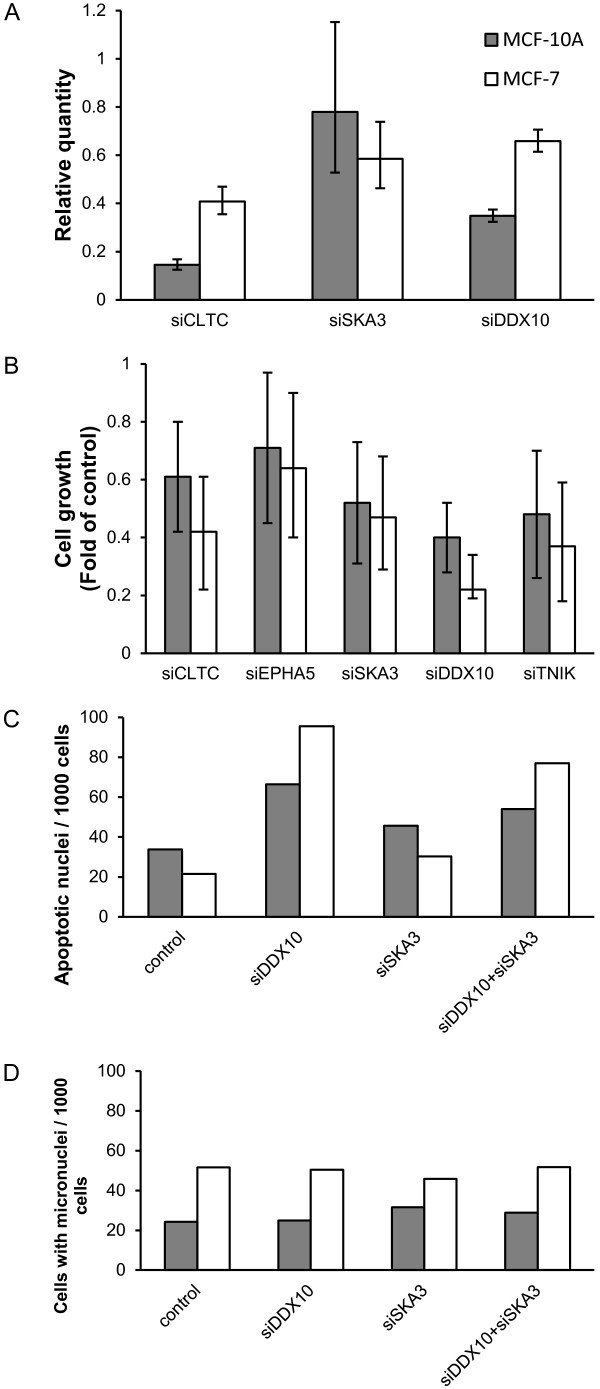
**Gene knockdown results in cell growth inhibition and suppression of *DDX10 *leads to increased apoptosis.** (**A**) Realtime quantitative PCR post-transfection of esiRNAs targeting *CLTC*, *SKA3* and *DDX10* showed efficient suppression of these three genes at the mRNA level. Suppression of *EPHA5* and *TNIK* was not able to be assessed using this technique due to poor quality of primers. (**B**) siRNA targeting *CLTC*, *EPHA5*, *SKA3*, *DDX10* and *TNIK* transfected MCF-10A and MCF-7 cell growth *in vitro* relative to controls 70 h and 120 h, respectively, after transfection is reported. Data from two independent experiments are shown with error bars representing standard deviations. Apoptosis (**C**) and micronuclei formation (**D**) of cell lines after transfection with siRNA targeting *DDX10* (MCF-10A, N = 2486; MCF-7, N = 2838), *SKA3* (MCF-10A, N = 2823; MCF-7, N = 5035) or both siRNAs (MCF-10A, N = 2390; MCF-7, N = 4701) was shown by numbers of cell nuclei that exhibit apoptotic nuclear morphology and micronuclei, respectively, per 1000 cells. siGFP transfected cells served as controls (MCF-10A, N = 2638; MCF-7, N = 3896). Data from a representative experiment.

In order to gain insight into the functional role of some of these genes in tumor cell growth, we performed small interfering RNA (siRNA) knock-down analysis targeting candidate genes *CLTC* (clathrin, heavy chain), *EPHA5* (EPH receptor A5), *SKA3* (spindle and kinetochore associated complex subunit 3), *DDX10* (DEAD (Asp-Glu-Ala-Asp) box polypeptide 10) and *TNIK* (TRAF2 and NCK interacting kinase). We transfected siRNA targeting each gene into human breast adenocarcinoma cell line MCF-7 and the mammary epithelial cell line MCF-10A. Downregulation of *CLTC*, *SKA3* and *DDX10* expression was confirmed by RT-PCR in both cell lines (Figure 2A). However, we failed to evaluate the effectiveness of knock-down for genes *EPHA5* and *TNIK* due to poor quality of primers. Relative cell growth (fold of siGFP-transfected control) was 0.42 ± 0.2, 0.64 ± 0.24, 0.47 ± 0.18, 0.22 ± 0.03 and 0.37 ± 0.19 in *CLTC*, *EPHA5*, *SKA3*, *DDX10* and *TNIK* knock-down MCF-7 cell lines, respectively. Relative cell growth in transfected MCF-10A cell lines was 0.61 ± 0.19, 0.71 ± 0.26, 0.52 ± 0.21, 0.4 ± 0.12 and 0.48 ± 0.22 for *CLTC*, *EPHA5*, *SKA3*, *DDX10* and *TNIK* respectively. Suppression of any of these genes led to growth inhibition in both cell lines tested (Figure 2B).

Consistent with previous studies [2,6,12], we did not observe frequently recurrent rearrangements. The only events that disrupted the same genes in two tumors were translocations t(11:13)(q22.3,q12.11), which breakpoints were located within the genes *SKA3* and *DDX10*. *SKA3* is required for spindle checkpoint silencing, the maintenance of chromosome cohesion in mitosis and metaphase to anaphase progression [18,19], whereas *DDX10* encodes a DEAD-box RNA helicase and is known to form an *NUP98*-*DDX10* fusion oncogene in leukemia [20]. In addition to decreased cell growth, we observed a higher percentage of cells with apoptotic nuclear morphology after suppression of *DDX10* expression in these MCF-7 (95 apoptotic nuclei in *DDX10*-suppression vs. 22 in control, per 1000 cells) and MCF-10A (66 nuclei in *DDX10*-suppression vs. 34 in control, per 1000 cells) cell line. In cells treated with siRNA targeting *SKA3*, apoptotic nuclei were observed at a similar or slightly greater frequency compared to control (Figure 2C). We also investigated the formation of micronuclei. Consistent with previous observations [21,22], about 5% of MCF-7 cells and 2-3% MCF-10A cells harbored micronuclei, while suppression of either *DDX10* or *SKA3* expression did not lead to any significant change in this frequency (Figure 2D). These findings indicate that the *SKA3*/*DDX10* alterations may have potential roles in tumor development, and *DDX10* may be involved in pathways mediating cell apoptosis.

We also observed and validated a putative in-frame gene fusion of *PLEKHA7* and *ASIC2* as a result of a translocation between chromosomes 11 and 17. This rearrangement was constitutional rather than somatic. Nevertheless, we cannot exclude the intriguing possibility that this fusion could be driving tumorigenesis, since somatic point mutations and rearrangements in *ASIC2* have been observed in previous studies [9,12,23].

## Discussion

### Long-insert mate pair sequencing for detecting gene rearrangements

In this study we chose to perform long-insert (~2.5 kb) mate pair sequencing to comprehensively identify structural alterations in receptor-negative breast cancers. Potential advantages of the approach include higher sensitivity and higher likelihood of detecting SVs within repetitive regions. Long insert lengths also reduce the need for high sequence coverage, especially when searching for potential breakpoints in the chromosomes as consequences of SVs. However, one possible drawback of long inserts could arguably be an increased difficulty of validation, since PCR product sizes may be prohibitively large when few reads span a breakpoint.

In this study, the majority (65%) of validated rearrangements were interchromosomal, which underly the fact that most (83%) true deletions were confirmed to be germline variants instead of somatic events, whereas a smaller number of translocations (35%) were present in matched normal tissues. However, this proportion differs from a previous investigation where interchromosomal events only composed less than 10% of all kinds of structural variations. Despite the limited sample size in both studies and different classification system of rearrangements, the ratios of interchromosomal events to intrachromosomal deletions and inversions in these two studies vary to a large extent: 1.86 in our study and 0.38 in [12]. This discrepancy may be explained by the selection of rearrangements for validation in this study, since we only attempted to confirm the SVs that occurred in or within two insert lengths of RefSeq genes and within two insert lengths of similar SVs in other tumors. It might also indicate the difference in the detectability of distinct types of rearrangement between these two studies due to approaches used (i.e. insert size of genomic library, software for variant calling, etc.).

### Potential function in cell growth and survival revealed by siRNA knock-down analysis

Results of siRNA experiments in cell lines indicate that the genes *CLTC*, *EPHA5*, *SKA3*, *DDX10* and *TNIK* might be functional in cell growth, and *DDX10* is probably involved in cell apoptosis. However, the cell lines MCF-7 and MCF-10A used in this analysis may be suboptimal since they do not represent receptor negative breast cancers. Therefore, additional cell lines, especially receptor negative breast cancer cell lines will need to be studied to ultimately determine the function of these genes in breast cancer development.

### Recurrently affected genes in other cancer genome sequencing studies

To identify potential recurrent somatic rearrangements in breast cancer, we compared validated somatic SVs in this study with findings from several recently published breast cancer genome reports [2,4,6,11,12]. None of the validated SVs were observed in any other previous studies, demonstrating that recurrent somatic rearrangements are very rare in breast cancers. However, some genes were affected by somatic SVs in more than one breast cancer.

Somatic deletions in *EYA1* were previously reported in four ER + breast tumors and one TNBC [4,12], revealing a combined prevalence of 7% (6 affected cases in a total of 85 samples investigated). *EYA1* encodes a transcription factor where mutations have been associated with the branchio-oto-renal syndrome and other developmental abnormalities [24]. DSCAM was affected by complex SVs including amplification, inversion, deletion and interchromosomal translocation in three additional breast tumors [11,12]. *DSCAM* (Down syndrome cell adhesion molecule) activates JNK and p38 MAP kinases and is important for axon guidance in the central neuron system [25]. Amplification and interchromosomal translocation of *CLTC* encoding the heavy chain of clathrin, which is required for the function of the mitotic spindle [26], were reported in two breast tumors [4,12]. *CLTC* also forms fusion genes in 25-30% of lymphomas and myofibroblastic tumors [27]. Deletion, amplification and interchromosomal translocation of *KIAA1217* were previously discovered in two breast cancers [11,12]. Moreover, genes *EPHA5*, *LRBA* (LPS-responsive vesicle trafficking, beach and anchor containing), *THSD4* (thrombospondin, type I, domain containing 4), *DDX10*, *GUCY1A2* (guanylate cyclase 1, soluble, alpha 2), *JMJD1C* (jumonji domain containing 1C), *KDM3B* (lysine (K)-specific demethylase 3B), *KSR1* (kinase suppressor of Ras 1), *PFKM* (phosphofructokinase, muscle), *PIP4K2A* (phosphatidylinositol-5-phosphate 4-kinase, type II, alpha), *RORA* (RAR-related orphan receptor A), *SMARCC1* (SWI/SNF related, matrix associated, actin dependent regulator of chromatin, subfamily c, member 1), *SPRED2* (sprouty-related, EVH1 domain containing 2), *TMEM99* (transmembrane protein 99) and *TNIK* have been reported for deletions in at least one breast tumor in previous studies [4,11,12], suggesting that the roles of these genes in cancer merit further investigation. Interestingly, *JMJD1C* was observed downregulated in breast cancers compared to normal tissues, suggesting it as a potential tumor suppressor gene [28]. *LRBA* was found upregulated in several different cancers including ER + breast tumor, and *LRBA* knockdown promotes cancer cell apoptosis [29].

### Comparison of breakpoint location to array painting study and fragile sites

**Figure 3:**
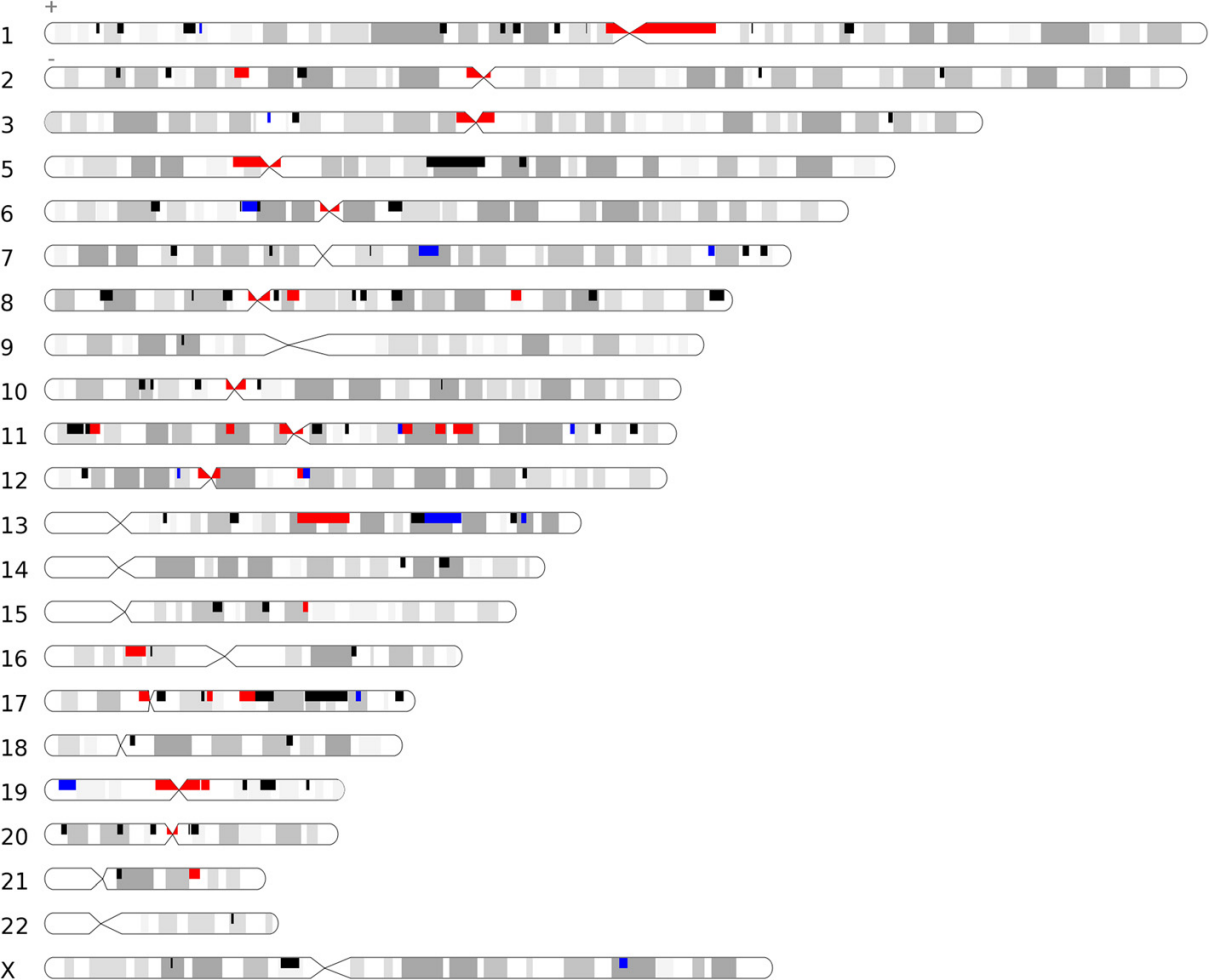
**Overlap of translocation breakpoints and array painting.** BAC regions studied by Howarth et al. are shown as boxes on the ideogram. Black boxes indicate regions not overlapping with breakpoints from this study, blue boxes indicate regions in which one breakpoint was observed in this study, and red boxes indicate regions that contain more than one breakpoint. The gray shadows represent chromosome bands.

We also compared the translocation breakpoints in the present study to the total non-redundant translocation breakpoint regions (456615397 bp) previously revealed by array painting of three breast cancer cell lines HCC1806, HCC1187 and ZR-75-30 [30]. Of our 2816 breakpoints in 1408 translocations, we observe 38% within the translocation breakpoint regions, significantly higher than the expected 15% (p < 1*10^-194^; binomial test). Out of 158 non-redundant translocation breakpoint regions, 52 co-occur at least once with putative translocations in this study (Figure 3), altogether suggesting a correlation in breakpoint regions of translocations in breast cancers between our study and the previous screen, which might reveal two possible scenarios; first, common defects in the DNA repair mechanisms in breast cancer may led to similar patterns of chromosome breakage, or second, the breakpoints represent driver rearrangements conferring a selective advantage in carcinogenesis.

We also studied the overlap between the translocation breakpoints in this study and the fragile sites of chromosomes (402989448 bp) previously reported by Debacker and coworkers [31]. We observed 11% of total breakpoints occur within the fragile regions, as compared to the expected 13.4%, which suggests that there is no significant correlation between double strand breaks and fragile sites (binomial test). Therefore, the possibility that the correlation between mate pair sequencing and array painting is caused by intrinsic characteristics of chromosomes could be ruled out.

### Enrichment of consensus cancer genes in genes affected by rearrangements

While the mechanisms and routes to breast cancer may be complex, the accumulation of sequenced tumors will eventually lead to a clearer understanding of the necessary genetic rearrangements. This work focuses on 15 samples, and we identify alterations that recur not only within our samples, but also with previous large scale studies [6,9,10,12]. Moreover, among the 29 genes disrupted directly by SVs, 4 genes are included in the human cancer-gene census [32]: *CHN1*, *CLTC*, *DDX10* and *MECOM*, suggesting an enrichment of consensus cancer genes in our results (binomial test; p < 0.0001). However, it should be noted that these genes may not represent all the genes affected by the rearrangements, since only the validated SVs were considered here and we only attempted to validate deletions, inversions and translocations that occurred in or near RefSeq genes.

The distribution of SVs varies markedly, reflecting the unique genetic composition of each tumor. However, the most striking difference is in the number of insertions, where two samples (120 T and 150 T) have thousands of insertions, in contrast to much lower numbers in other samples (Additional file 6). This does not seem to be the consequence of differences in sequence coverage, since the two most deeply sequenced samples harbor only 67 insertions in total. *BRCA1* and *BRCA2* mutational analyses detect no germline mutations in the two patients with extremely large numbers of insertions, indicating that this feature is not caused by deficiency in DNA repair mechanisms as a result of *BRCA1* or *BRCA2* mutations. The bulk of the insertions in samples 120 T and 150 T tend to be shorter with an average insertion length of roughly 600 bp, compared to about 1000 bp in other samples (Additional file 7 and Additional file 8), which may suggest a different mutagenesis mechanism in these two samples.

## Conclusions

In this study we identify gene rearrangements in receptor negative breast cancer genomes using long-insert whole-genome mate pair sequencing. Somatic rearrangements disrupting genes composed by both known cancer genes and genes not previously correlated with cancer have been validated. These genes include epigenetic regulators, genes involved in mitosis and multiple signaling pathways and other genes whose functions are largely unknown. Consistent with previous studies [12], we did not observe frequently recurrent rearrangements, which verify the fact that breast cancer is a highly heterogeneous disease that a large number of low-frequency rearrangements may synergistically contribute to its development.

## Methods

### Sample handling, DNA library construction and mate pair sequencing

The study was approved by the Regional Ethical Review Board of Uppsala (2007/116). Fifteen breast cancer specimens with paired DNA from adjacent normal breast tissue derived from a part of the breast resectate that was devoid of macroscopic tumor were analyzed. Tumor cellularity was more than 50% in all the tumor samples while the normal tissues were confirmed not containing any tumor cells by microscopic inspection by a pathologist. Two out of the 15 patients had previous malignancies in the ovary or cervix, respectively. All cancer samples showed negative staining of hormone receptors ER and PR, whereas three of the 15 samples exhibited overexpression of HER2, determined by IHC (Additional file 1). Genomic DNA was extracted from SDS-Proteinase K digested fresh frozen tissues by phenol-chloroform. Qualification and quantification of DNA was carried out by NanoDrop (Thermo Scientific) and real-time PCR, respectively. *BRCA1* and *BRCA2* mutation analysis was performed by PCR followed by Sanger sequencing of all protein coding regions of the two genes in normal DNA samples.

Thirty μg of DNA from each sample were used to construct SOLiD3 or SOLiD4 mate-pair libraries according to the manufacturer’s instructions. Briefly, the DNA was sheared into fragments of about 2,500 bp by HydroShear (Genomic Solutions) and end-repaired using End Polishing Enzyme 1 and 2. Cap adaptors (5^′^-pACAGCAG-3^′^, 5^′^-CATGTCGTCp-3^′^) are ligated to both ends of the fragments. Next, the adapter ligated DNA sample was separated on a 0.8% agarose gel and DNA fragments of about 2,500 bp in length were recovered and purified. The sizes and concentrations of adapter ligated DNA strands were quantified using a Bioanalyzer kit (DNA 7500, Agilent). The samples were circularized using a biotinylated internal adaptor, nick-translated with *E*.*coli* DNA polymerase 1 and digested with T7 exonuclease and S1 nuclease. Digested DNA was end-repaired using End Polishing Enzyme 1 and 2 and bound to streptavidin beads. P1 (5^′^-CCACTACGCCTCCGCTTTCCTCTCTATGGGCAGTCGGTGAT-3^′^, 5^′^-ATCACCGACTGCCCATAGAGAGGAAAGCGGAGGCGTAGTGGTT-3^′^) and P2 adaptors (5^′^-AGAGAATGAGGAACCCGGGGCAGTT-3^′^, 5^′^-CTGCCCCGGGTTCCTCATTCTCT-3^′^) were ligated to the fragments. The libraries were further nick-translated followed by PCR-based amplification and released from the beads. PCR products were separated on a 4% agarose gel and the 250–350 bp library bands were recovered, purified, and verified using a Bioanalyzer kit (Agilent, DNA 1000). Throughout the library preparation procedure, DNA was purified and concentrated with QIAquick columns (QIAGEN) after each enzymatic reaction and PCR. Emulsion PCR was performed according to the manufacturer's manual (SOLiD3 System Templated Bead Preparation Guide, Life Technologies) before SOLiD sequencing. Subsequently, 50 base pairs from each end were collected on the Life Technologies SOLiD3 or SOLiD4 instrument. Genotype data have been deposited at the European Genome-phenome Archive (EGA, http://www.ebi.ac.uk/ega/), which is hosted by the European Bioinformatics Institute, under accession number EGAS00001000438.

### Sequence alignment and rearrangement detection

The resulting reads were mapped to the human genome (HG18) using Corona Lite (Applied Biosystems). All reads with ambiguous paired mappings and all redundant pairs were removed. The insert lengths between paired ends were compared to the corresponding distances between their alignments against the reference genome in order to detect indels, and inversions were detected by disparate strand orientations of paired alignments. Similarly, fragments whose ends mapped against different chromosomes may suggest inter-chromosomal rearrangements such as translocations or transpositions of DNA between chromosomes. Fragments were greedily clustered if they report the same type of rearrangement at the same chromosomal position, resulting in predicted structural variations (SVs). To filter out spurious rearrangements, first, the SVs which were also observed in control samples from two healthy individuals (previously reported in [33]) were removed. Second, we removed all events situated within two insert lengths from telomeric or centromeric regions, or known gaps in the reference genome. Third, known variations based on the Database of Genomic Variants [34] were removed. This process removed on average 89% of putative somatic SVs (Additional file 2 and Additional file 9). Finally, for insertions and translocations, we analyzed the positioning of anchors versus the reference genome. Essentially, we assumed that a genuine translocation or transposition is characterized by a correlation in the positions of mate-paired anchors; as the upstream anchor position increases, so should the downstream anchor position. In the case of an inversion, we expect an inverse relationship between the upstream and downstream anchor. In terms of correlation between upstream position and downstream position, we expect a strong and significant positive correlation between up- and downstream anchors in case of a same-orientation translocation while a strong and significant negative correlation between anchors is expected in case of an inverted translocation. We therefore calculated the correlation coefficient between anchor positions on each chromosome in order to further exclude false positives caused by repetitive sequences from true positive inter-chromosomal rearrangements. Translocations with significant positive or negative correlation coefficients were considered more likely to be true positives. A detailed study of the statistical properties of translocations has been carried out (Hooper et al., submitted). All genome coordinates of rearrangements were converted to the latest human genome version HG19 for the readers’ convenience by LiftOver (http://genome.ucsc.edu/cgi-bin/hgLiftOver).

### Rearrangement validation by PCR and capillary sequencing

Rearrangements were selected for validation if they fulfill all of the three criteria: (1) occured in or within two insert lengths of RefSeq genes, (2) were supported by at least four reads, and (3) occured within two insert lengths of similar rearrangements in other tumor samples. Exception applied to the validation of translocations in the two deeply sequenced samples (113 T and 114 T), in which the cutoff of supporting reads were set to 40 instead of 4 mate pairs and only those with significant anchor correlation were attempted for confirmation (Hooper et al., submitted). We furthermore consider the impact and interest of rearrangements to increase with read support, since it may reflect both a higher degree of accuracy and proportion of the total tumor population. Additionally, primer design is more accurate due to larger target sizes.

To validate selected putative rearrangements, we designed forward and reverse PCR primers within a 200-bp range as close as possible to the breakpoints using Primer3 [35] and verified against the human reference genome (hg18) using Bowtie [36]. Primers were discarded if they had multiple matches to the reference or predicted to yield too large PCR products. This approach minimizes the expected PCR product size and increases the chance of detection. Primers were based on the DNA on either side of a breakpoint, and were not allowed to be within the reads they were designed to validate. For each chosen deletion, inversion and translocation, up to 5 different primer pairs were evaluated in PCR to enhance detectability. Insertions could not be validated directly by PCR due to the large product sizes. PCR for each selected candidate rearrangement was carried out on both tumor DNA and patient-matched normal DNA in parallel to determine whether the rearrangements were somatic. The PCR products of somatic rearrangements were further purified and analyzed by capillary sequencing in order to determine the exact breakpoints and those that obtain sequences covering the rearrangement breakpoints were considered as validated somatic rearrangements.

### RNA interference in normal and malignant breast cells

MCF-7 cells were cultured in McCoys 5A (Gibco) supplemented with 10% FBS (Gibco) and penicillin-streptomycin (100 μg/ml each, Gibco). MCF-10A cells were cultured in phenol red-free DMEM-F12 media (Gibco) supplemented with 5% heat-inactivated horse serum (Gibco), hydrocortisone (0.5 μg/ml, Sigma-Aldrich), insulin (10 μg/ml, Sigma-Aldrich), epidermal growth factor (0.02 ng/ml, PeproTech), cholera toxin (0.1 μg/ml, Sigma-Aldrich) and penicillin-streptomycin (100 μg/ml each, Gibco). Mission endoribonuclease-prepared small interfering RNA (esiRNA) targeting *CLTC*, *DDX10*, *EPHA5*, *SKA3* and *TNIK* were obtained from Sigma-Aldrich. MCF-7 and MCF-10A cells were transfected using Lipofectamine 2000 (Invitrogen, Carlsbad, CA, USA). GFP specific siRNA (AACUUCAGGGUCAGCUUGC) was used as a control. In order to measure the efficiency of depletion, total RNA was extracted using QIAamp RNA Blood Mini kit (Qiagen) 48 h after esiRNA transfection and the cDNA was generated from 1 μg total RNA with RevertAid H Minus First Strand cDNA Synthesis kit (Fermentas) according to the manufacturer’s instruction. Real-time quantitative PCR was done using an Applied Biosystems StepOne qPCR instrument (PCR conditions and primers are available upon request). Data analysis was performed using the software provided by Applied Biosystems Inc with β-actin as a reference gene. For cell growth assays, cells (100 000 cells/well for MCF-7 and 50 000 cells/well for MCF-10A) seeded in 12-well tissue culture plates were transfected 24 h later with esiRNA. MCF-7 and MCF-10A cell growth was determined in the Incucyte system (Essen Instruments, Ann Arbor, MI, USA) according to the manufacturer’s instruction 120 h and 70 h after transfection, respectively.

For investigation of micronuclei and apoptosis formation, cells (30 000 cells/well for MCF-7 and 18 000 cells/well for MCF-10A) were seeded in LabTekII 8 well chamber slides (Nunc). The cells were allowed to attach overnight and then transfected with esiRNA targeting *DDX10* and/or *SKA3*. After 24 h growth in the incubator, slides were fixed in 3.7% formaldehyde (Sigma-Aldrich) for 15 min. Cell nuclei were stained with Hoechst 33342 (1:10 000 in 1xPBS) for 40 min. Cells were imaged with a Zeiss AxioImager M2 fluorescence microscope. The total number of cells, the number of micronuclei and the number of apoptotic cell nuclei were determined manually from the images using the Cell Counter Plugin for ImageJ 1.45 s.

## Competing interests

The authors declare that they have no competing interests.

## Authors’ contributions

SDH carried out the bioinformatic and statistical analyses, performed primer design for PCR validation, participated in data interpretation and drafted the manuscript. XJ carried out the validation by PCR and sequencing, participated in data interpretation and drafted the manuscript. TD and CL carried out the RNA interference assays. FW and JB participated in annotation of clinical samples. CTR performed the sequencing data analyses. TS conceived and designed the study and helped to draft the manuscript. All authors read and approved the final manuscript.

## Supplementary Material

Additional file 1 Characteristics of the breast cancer samples. Click here for file

Additional file 2 Summary of sequencing and numbers of rearrangements discovered in each breast cancer genome. Click here for file

Additional file 3 Rearrangements detected in mate pair sequencing. Click here for file

Additional file 4 **Circos plots of somatic rearrangements in breast cancer genomes.** Outer histogram of the Circos plot displays the number of deletions in each bin, and the inner histogram displays predicted insertions. Connections represent PCR-validated (hashed lines) and sequence-validated (solid lines) somatic translocations. Click here for file

Additional file 5 Gene Ontology (GO) terms of affected genes. Click here for file

Additional file 6 **Insertions detected in 4 breast cancer samples.** Insertions were illustrated in two samples harboring insertions at much higher prevalence (120 T and 150 T) and two samples (149 T and 116 T) representing the others. Each inversion supported by at least four independent mate-pairs is illustrated as a red bar on its chromosome. Click here for file

Additional file 7 Numbers and sizes of deletions and insertions supported by at least three reads in breast cancer genomes. Click here for file

Additional file 8 Numbers and sizes of deletions and insertions supported by at least four reads in breast cancer genomes. Click here for file

Additional file 9 Flowchart of structural variants (SV) identification procedures. Click here for file
