# Supplementary material for: Gene rearrangements in hormone receptor negative breast cancers revealed by mate pair sequencing
Source: BMC Genomics. 2013 Mar 12;14:165. doi: 10.1186/1471-2164-14-165 (PMC3600027; doi:10.1186/1471-2164-14-165)
Supplement: Additional file 1 — Characteristics of the breast cancer samples. [file 1471-2164-14-165-S1.pdf]

Additional file 1 - Characteristics of the breast cancer samples.

Sample ID	Age	PAD[a]	ER[b]	PR[b]	HER2[b]	Elston grade	Positive nodes	Total nodes	Ploidy[c]	Prev malign[d]
113T	51	Ductal breast carcinoma	-	-	+	3+3+3			D	No
114T	55	Ductal breast carcinoma + DCIS	-	-	+	3+3+3	19	21	T	Cervix
116T	45	Ductal carcinoma	-	-	-	3+3+3	0	12	A	No
117T	46	Ductal breast carcinoma + DCIS	-	-	+	3+3+3			A	No
118T	51	Ductal breast carcinoma + DCIS	-	-	-	3+3+2			D	No
119T	49	Ductal breast carcinoma	-	-	-	3+3+3	1	6	D	No
120T	60	Ductal breast carcinoma + DCIS	-	-	-	3+3+3	0	12	T	Ovary
147T	42	Atypical medullary breast carcinoma	-	-	-	3+3+3			A	No
148T	61	Ductal breast carcinoma	-	-	N/A	3+3+3			A	No
149T	65	Ductal breast carcinoma + DCIS	-	(+)	-	3+3+3			N/A	No
150T	41	Atypical medullary breast carcinoma + DCIS	(+)	-	-	3+3+3	3	6	A	No
151T	55	Ductal breast carcinoma	-	-	-	3+3+3	7	8	A	No
152T	54	Atypical medullary breast carcinoma	-	-	-	3+3+3			A	No
153T	52	Ductal breast carcinoma	-	-	-	3+3+2	0	13	A	No
154T	72	Ductal breast carcinoma	-	-	-	8			A	No

[a]PAD, pathological anatomical diagnosis.

[b]Estrogen receptor (ER), progesterone receptor (PR) and human epidermal receptor 2 (HER2) status: -, negative; +, positive; (+), borderline; N/A, data not available.

[c]Ploidy, D, diploid; T, triploid; A, aneuploid; N/A, not available.

[d]Malignancy prior to diagnosis of breast cancer.
