# Supplementary material for: Gene rearrangements in hormone receptor negative breast cancers revealed by mate pair sequencing
Source: BMC Genomics. 2013 Mar 12;14:165. doi: 10.1186/1471-2164-14-165 (PMC3600027; doi:10.1186/1471-2164-14-165)
Supplement: Additional file 2 — Summary of sequencing and numbers of rearrangements discovered in each breast cancer genome. [file 1471-2164-14-165-S2.pdf]

Additional file 2 - Summary of sequencing and numbers of rearrangements discovered in each breast cancer genome.

Sample ID	Clone coverage (fold)	Total mapped reads	Rearr[a]	Pre-filtering				Post-filtering				% remaining after filter			
				Ins[b]	Del[b]	Inv[b]	Trans[b]	Ins[b]	Del[b]	Inv[b]	Trans[b]	Ins[b]	Del[b]	Inv[b]	Trans[b]
113T	107.8	137772615	2.00%	173	1523	217	921	50	185	19	670	29%	12%	9%	73%
114T	65.1	83158392	7.10%	71	1152	203	753	17	172	58	521	24%	15%	29%	69%
116T	6.2	7927919	11.50%	1327	414	163	177	50	18	34	7	4%	4%	21%	4%
117T	4.5	5806942	8.00%	45	197	21	40	3	13	3	2	7%	7%	14%	5%
118T	6.9	8801269	4.50%	628	492	190	277	16	25	17	42	3%	5%	9%	15%
119T	6.4	8182333	12.30%	363	395	190	199	4	14	42	13	1%	4%	22%	7%
120T	6.9	8855683	15.40%	20507	372	144	187	3265	13	32	10	16%	3%	22%	5%
147T	6.5	8302519	3.20%	368	424	97	157	5	13	4	13	1%	3%	4%	8%
148T	8.9	11364407	8.00%	3607	885	293	260	260	32	63	29	7%	4%	22%	11%
149T	9.3	11938694	9.40%	2000	900	311	275	120	83	53	25	6%	9%	17%	9%
150T	9.6	12268933	10.80%	16413	684	187	220	2466	27	22	11	15%	4%	12%	5%
151T	6.6	8451408	5.70%	606	548	94	132	11	14	3	1	2%	3%	3%	1%
152T	6.1	7785332	5.80%	645	453	95	125	14	13	2	4	2%	3%	2%	3%
153T	9.8	12482817	6.20%	1190	688	249	317	87	59	45	33	7%	9%	18%	10%
154T	9.8	12567751	4.70%	1073	612	231	219	50	43	46	27	5%	7%	20%	12%

[a]Proportion of reads reporting rearrangements.

[b]Numbers of putative rearrangements, Ins, insertions; Del, deletions; Inv, inversions; Trans, interchromosomal translocations.
