# Supplementary figures and images for: Gene rearrangements in hormone receptor negative breast cancers revealed by mate pair sequencing
Source: BMC Genomics. 2013 Mar 12;14:165. doi: 10.1186/1471-2164-14-165 (PMC3600027; doi:10.1186/1471-2164-14-165)

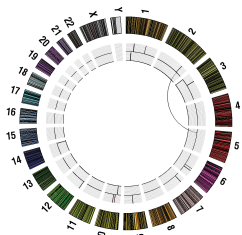

116T

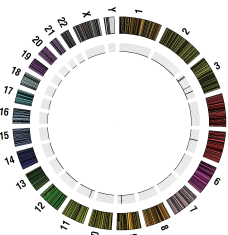

117T

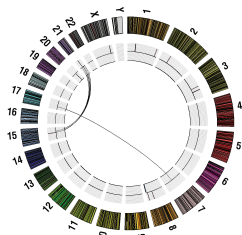

118T

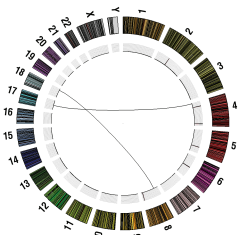

119T

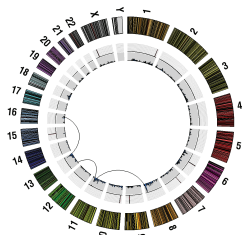

120T

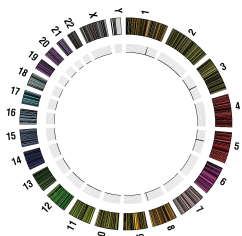

147T

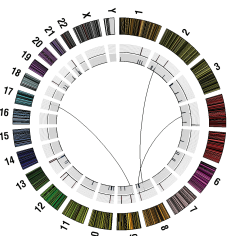

148T

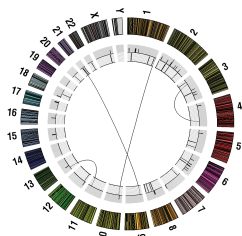

149T

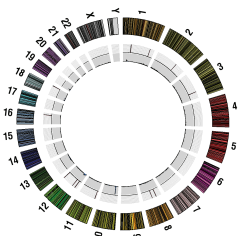

150T

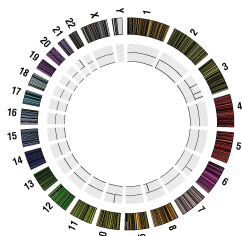

151T

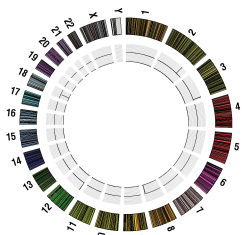

152T

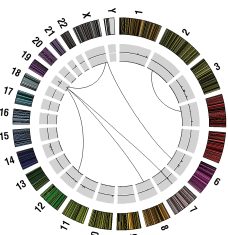

153T

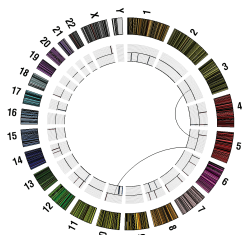

154T

Supplement: Additional file 4 — Circos plots of somatic rearrangements in breast cancer genomes. Outer histogram of the Circos plot displays the number of deletions in each bin, and the inner histogram displays predicted insertions. Connections represent PCR-validated (hashed lines) and sequence-validated (solid lines) somatic translocations. [file 1471-2164-14-165-S4.pdf]

Chromosome

120T

150T

149T

116T

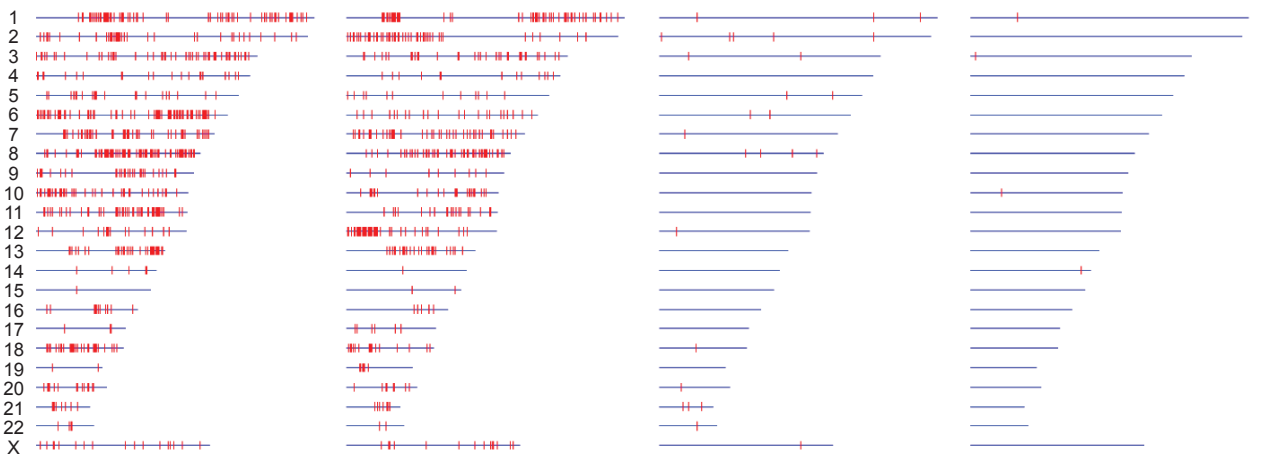

Position (Mb)

Supplement: Additional file 6 — Insertions detected in 4 breast cancer samples. Insertions were illustrated in two samples harboring insertions at much higher prevalence (120 T and 150 T) and two samples (149 T and 116 T) representing the others. Each inversion supported by at least four independent mate-pairs is illustrated as a red bar on its chromosome. [file 1471-2164-14-165-S6.pdf]
