# Supplementary material for: Gene rearrangements in hormone receptor negative breast cancers revealed by mate pair sequencing
Source: BMC Genomics. 2013 Mar 12;14:165. doi: 10.1186/1471-2164-14-165 (PMC3600027; doi:10.1186/1471-2164-14-165)
Supplement: Additional file 5 — Gene Ontology (GO) terms of affected genes. [file 1471-2164-14-165-S5.pdf]

Additional file 5. Gene Ontology (GO) terms of affected genes.

Gene	GO ID	GO term
**Epigenetic regulators**		
JMJD1C	GO:0016568	chromatin modification
	GO:0006355	regulation of transcription, DNA-dependent
	GO:0006351	transcription, DNA-dependent
	GO:0007596	blood coagulation
	GO:0046872	metal ion binding
	GO:0005654	nucleoplasm
	GO:0016702	oxidoreductase activity, acting on single donors with incorporation of molecular oxygen, incorporation of two atoms of oxygen
EYA1	GO:0016576	histone dephosphorylation
	GO:0006351	transcription, DNA-dependent
	GO:0045944	positive regulation of transcription from RNA polymerase II promoter
	GO:0009653	anatomical structure morphogenesis
	GO:0035909	aorta morphogenesis
	GO:0001658	branching involved in ureteric bud morphogenesis
	GO:0045165	cell fate commitment
	GO:0034613	cellular protein localization
	GO:0090103	cochlea morphogenesis
	GO:0005737	cytoplasm
	GO:0006302	double-strand break repair
	GO:0048704	embryonic skeletal system morphogenesis
	GO:0000132	establishment of mitotic spindle orientation
	GO:0035088	establishment or maintenance of apical/basal cell polarity
	GO:0060487	lung epithelial cell differentiation
	GO:0046872	metal ion binding
	GO:0001656	metanephros development
	GO:0042474	middle ear morphogenesis
	GO:0043066	negative regulation of apoptotic process
	GO:0005634	nucleus
	GO:0071600	otic vesicle morphogenesis
	GO:0042473	outer ear morphogenesis
	GO:0003151	outflow tract morphogenesis
	GO:0007389	pattern specification process
	GO:0060037	pharyngeal system development
	GO:0045739	positive regulation of DNA repair
	GO:0045747	positive regulation of Notch signaling pathway
	GO:0050679	positive regulation of epithelial cell proliferation
	GO:0072513	positive regulation of secondary heart field cardioblast proliferation
	GO:0016925	protein sumoylation
	GO:0004725	protein tyrosine phosphatase activity
	GO:0045664	regulation of neuron differentiation
	GO:0010212	response to ionizing radiation
	GO:0048752	semicircular canal morphogenesis
	GO:0007605	sensory perception of sound
	GO:0014706	striated muscle tissue development
SMARCC1	GO:0006338	chromatin remodeling
	GO:0003682	chromatin binding
	GO:0006351	transcription, DNA-dependent
	GO:0045944	positive regulation of transcription from RNA polymerase II promoter
	GO:0045893	positive regulation of transcription, DNA-dependent
	GO:0006357	regulation of transcription from RNA polymerase II promoter
	GO:0003713	transcription coactivator activity
	GO:0003677	DNA binding
	GO:0016514	SWI/SNF complex
	GO:0071778	WINAC complex
	GO:0001741	XY body

	GO:0008286	insulin receptor signaling pathway
	GO:0071565	nBAF complex
	GO:0007399	nervous system development
	GO:0071564	npBAF complex
	GO:0006337	nucleosome disassembly
	GO:0009887	organ morphogenesis
KDM3B	GO:0016568	chromatin modification
	GO:0006355	regulation of transcription, DNA-dependent
	GO:0006351	transcription, DNA-dependent
	GO:0046872	metal ion binding
	GO:0005634	nucleus
	GO:0016702	oxidoreductase activity, acting on single donors with incorporation of molecular oxygen, incorporation of two atoms of oxygen
H2AFY	GO:0016568	chromatin modification
	GO:0003682	chromatin binding
	GO:0000793	condensed chromosome
	GO:0001740	Barr body
	GO:0003677	DNA binding
	GO:0007549	dosage compensation
	GO:0000786	nucleosome
	GO:0006334	nucleosome assembly
**Mitosis**		
SKA3	GO:0007067	mitosis
	GO:0031110	regulation of microtubule polymerization or depolymerization
	GO:0005876	spindle microtubule
	GO:0007059	chromosome segregation
	GO:0000940	condensed chromosome outer kinetochore
	GO:0051301	cell division
	GO:0005737	cytoplasm
CLTC	GO:0007067	mitosis
	GO:0005819	spindle
	GO:0019886	antigen processing and presentation of exogenous peptide antigen via MHC class II
	GO:0007411	axon guidance
	GO:0016044	cellular membrane organization
	GO:0030118	clathrin coat
	GO:0030132	clathrin coat of coated pit
	GO:0030130	clathrin coat of trans-Golgi network vesicle
	GO:0030669	clathrin-coated endocytic vesicle membrane
	GO:0005829	cytosol
	GO:0007173	epidermal growth factor receptor signaling pathway
	GO:0006886	intracellular protein transport
	GO:0042470	melanosome
	GO:0005739	mitochondrion
	GO:0042059	negative regulation of epidermal growth factor receptor signaling pathway
	GO:0048011	nerve growth factor receptor signaling pathway
	GO:0005886	plasma membrane
	GO:0006892	post-Golgi vesicle-mediated transport
	GO:0031623	receptor internalization
	GO:0005198	structural molecule activity
	GO:0032588	trans-Golgi network membrane
	GO:0033572	transferrin transport
**Signaling pathways**		
TNIK	GO:0016055	Wnt receptor signaling pathway
	GO:0007256	activation of JNKK activity
	GO:0005083	small GTPase regulator activity
	GO:0005524	ATP binding
	GO:0031532	actin cytoskeleton reorganization
	GO:0005737	cytoplasm
	GO:0005856	cytoskeleton

	GO:0005634<br>GO:0046777<br>GO:0004674<br>GO:0055037<br>GO:0048814	nucleus<br>protein autophosphorylation<br>protein serine/threonine kinase activity<br>recycling endosome<br>regulation of dendrite morphogenesis
KSR1	GO:0007265<br>GO:0005524<br>GO:0005789<br>GO:0005622<br>GO:0046872<br>GO:0004672<br>GO:0004674	Ras protein signal transduction<br>ATP binding<br>endoplasmic reticulum membrane<br>intracellular<br>metal ion binding<br>protein kinase activity<br>protein serine/threonine kinase activity
EPHA5	GO:0019933<br>GO:0032314<br>GO:0005524<br>GO:0005004<br>GO:0030424<br>GO:0007411<br>GO:0030425<br>GO:0060997<br>GO:0009897<br>GO:0021766<br>GO:0005887<br>GO:0043025<br>GO:0048471<br>GO:0032793<br>GO:0032956<br>GO:0061178<br>GO:0005791<br>GO:0005005	cAMP-mediated signaling<br>regulation of Rac GTPase activity<br>ATP binding<br>GPI-linked ephrin receptor activity<br>axon<br>axon guidance<br>dendrite<br>dendritic spine morphogenesis<br>external side of plasma membrane<br>hippocampus development<br>integral to plasma membrane<br>neuronal cell body<br>perinuclear region of cytoplasm<br>positive regulation of CREB transcription factor activity<br>regulation of actin cytoskeleton organization<br>regulation of insulin secretion involved in cellular response to glucose stimulus<br>rough endoplasmic reticulum<br>transmembrane-ephrin receptor activity
CHN1	GO:0048013<br>GO:0007264<br>GO:0005096<br>GO:0032314<br>GO:0051056<br>GO:0005829<br>GO:0046872<br>GO:0008045<br>GO:0043547<br>GO:0050770	ephrin receptor signaling pathway<br>small GTPase mediated signal transduction<br>GTPase activator activity<br>regulation of Rac GTPase activity<br>regulation of small GTPase mediated signal transduction<br>cytosol<br>metal ion binding<br>motor neuron axon guidance<br>positive regulation of GTPase activity<br>regulation of axonogenesis
SPRED2	GO:0000188<br>GO:0030291<br>GO:0007275<br>GO:0010801<br>GO:0043517<br><br>GO:0090311<br>GO:0030658	inactivation of MAPK activity<br>protein serine/threonine kinase inhibitor activity<br>multicellular organismal development<br>negative regulation of peptidyl-threonine phosphorylation<br>positive regulation of DNA damage response, signal transduction by p53 class mediator<br>regulation of protein deacetylation<br>transport vesicle membrane
GUCY1A2	GO:0007165<br>GO:0035556<br>GO:0005525<br>GO:0007596<br>GO:0005829<br>GO:0004383<br>GO:0020037<br>GO:0030828	signal transduction<br>intracellular signal transduction<br>GTP binding<br>blood coagulation<br>cytosol<br>guanylate cyclase activity<br>heme binding<br>positive regulation of cGMP biosynthetic process
**Others**		
DDX10	GO:0005524<br>GO:0008026<br>GO:0003723	ATP binding<br>ATP-dependent helicase activity<br>RNA binding

	GO:0003724	RNA helicase activity
MECOM	GO:0003677	DNA binding
	GO:0006915	apoptotic process
	GO:0030154	cell differentiation
	GO:0071425	hemopoietic stem cell proliferation
	GO:0046329	negative regulation of JNK cascade
	GO:0043069	negative regulation of programmed cell death
	GO:0045892	negative regulation of transcription, DNA-dependent
	GO:0016607	nuclear speck
	GO:0045893	positive regulation of transcription, DNA-dependent
	GO:0051726	regulation of cell cycle
	GO:0003700	sequence-specific DNA binding transcription factor activity
	GO:0006351	transcription, DNA-dependent
	GO:0008270	zinc ion binding
METAP1D	GO:0031365	N-terminal protein amino acid modification
	GO:0004177	aminopeptidase activity
	GO:0046872	metal ion binding
	GO:0008235	metalloexopeptidase activity
	GO:0005739	mitochondrion
	GO:0018206	peptidyl-methionine modification
	GO:0006508	proteolysis
DSCAM	GO:0030424	axon
	GO:0007155	cell adhesion
	GO:0048813	dendrite morphogenesis
	GO:0070593	dendrite self-avoidance
	GO:0005576	extracellular region
	GO:0030426	growth cone
	GO:0005887	integral to plasma membrane
	GO:0007626	locomotory behavior
	GO:0007162	negative regulation of cell adhesion
	GO:0048842	positive regulation of axon extension involved in axon guidance
	GO:0042327	positive regulation of phosphorylation
	GO:0060060	post-embryonic retina morphogenesis in camera-type eye
PFKM	GO:0003872	6-phosphofructokinase activity
	GO:0005945	6-phosphofructokinase complex
	GO:0005524	ATP binding
	GO:0016324	apical plasma membrane
	GO:0006002	fructose 6-phosphate metabolic process
	GO:0070061	fructose binding
	GO:0006096	glycolysis
	GO:0046872	metal ion binding
	GO:0046716	muscle cell homeostasis
	GO:0051259	protein oligomerization
	GO:0044281	small molecule metabolic process
LRBA	GO:0005794	Golgi apparatus
	GO:0016023	cytoplasmic membrane-bounded vesicle
	GO:0005783	endoplasmic reticulum
	GO:0016021	integral to membrane
	GO:0005764	lysosome
	GO:0005886	plasma membrane
PCBP3	GO:0003677	DNA binding
	GO:0003723	RNA binding
	GO:0005829	cytosol
	GO:0016071	mRNA metabolic process
	GO:0005739	mitochondrion
	GO:0005634	nucleus
	GO:0030529	ribonucleoprotein complex
PIP4K2A	GO:0016308	1-phosphatidylinositol-4-phosphate 5-kinase activity
	GO:0016309	1-phosphatidylinositol-5-phosphate 4-kinase activity
	GO:0005524	ATP binding

	GO:0005829	cytosol
	GO:0035855	megakaryocyte development
	GO:0005634	nucleus
	GO:0006661	phosphatidylinositol biosynthetic process
	GO:0005886	plasma membrane
	GO:0044281	small molecule metabolic process
RCAN1	GO:0003677	DNA binding
	GO:0008015	blood circulation
	GO:0019722	calcium-mediated signaling
	GO:0007417	central nervous system development
	GO:0005737	cytoplasm
	GO:0051151	negative regulation of smooth muscle cell differentiation
	GO:0005634	nucleus
	GO:0043666	regulation of phosphoprotein phosphatase activity
	GO:0043627	response to estrogen stimulus
	GO:0009612	response to mechanical stimulus
	GO:0003700	sequence-specific DNA binding transcription factor activity
	GO:0007165	signal transduction
	GO:0048741	skeletal muscle fiber development
RORA	GO:0046068	cGMP metabolic process
	GO:0021702	cerebellar Purkinje cell differentiation
	GO:0004879	ligand-activated sequence-specific DNA binding RNA polymerase II transcription factor activity
	GO:0006809	nitric oxide biosynthetic process
	GO:0005654	nucleoplasm
	GO:0045944	positive regulation of transcription from RNA polymerase II promoter
	GO:0043030	regulation of macrophage activation
	GO:0043565	sequence-specific DNA binding
	GO:0003707	steroid hormone receptor activity
	GO:0006367	transcription initiation from RNA polymerase II promoter
	GO:0008270	zinc ion binding
KIAA1217	GO:0005737	cytoplasm
	GO:0048706	embryonic skeletal system development
THSD4	GO:0004222	metalloendopeptidase activity
	GO:0005578	proteinaceous extracellular matrix
TMEM99	GO:0016021	integral to membrane
ZNF536	GO:0045665	negative regulation of neuron differentiation
	GO:0048387	negative regulation of retinoic acid receptor signaling pathway
	GO:0005634	nucleus
	GO:0006355	regulation of transcription, DNA-dependent
	GO:0044323	retinoic acid-responsive element binding
	GO:0006351	transcription, DNA-dependent
	GO:0008270	zinc ion binding
