# Supplementary material for: Gene rearrangements in hormone receptor negative breast cancers revealed by mate pair sequencing
Source: BMC Genomics. 2013 Mar 12;14:165. doi: 10.1186/1471-2164-14-165 (PMC3600027; doi:10.1186/1471-2164-14-165)
Supplement: Additional file 7 — Numbers and sizes of deletions and insertions supported by at least three reads in breast cancer genomes. [file 1471-2164-14-165-S7.pdf]

Additional file 7 - Numbers and sizes of deletions and insertions supported by at least three reads in breast cancer genomes.

Tumor sample	Deletion				Insertion			
	Total	Mean size (bp)	Variance (bp)	Median size (bp)	Total	Mean size (bp)	Variance (bp)	Median size (bp)
113T	185	975579	4567553	5744	50	1129	219	1161
114T	172	2733748	8666480	6980	17	1119	158	1105
116T	18	6440	6330	4795	50	959	270	1001
117T	13	11248605	32527069	6149	3	1148	460	1563
118T	25	1929248	5395084	5130	16	1151	280	1274
119T	15	35791	79493	5201	5	1167	378	1512
120T	13	275347	933007	5214	3265	634	194	614
147T	13	6080	2750	5173	5	1007	522	1370
148T	33	931470	3997911	4601	261	864	483	743
149T	83	270583	1224328	4604	120	1180	414	1244
150T	27	8017	18621	4079	2466	616	238	588
151T	14	1325992	4763543	4774	12	1225	227	1184
152T	13	2315489	5587721	4976	14	1367	297	1471
153T	59	16368	70042	4284	87	775	414	712
154T	43	9563	23550	4628	50	1326	372	1508
