# Supplementary material for: Gene rearrangements in hormone receptor negative breast cancers revealed by mate pair sequencing
Source: BMC Genomics. 2013 Mar 12;14:165. doi: 10.1186/1471-2164-14-165 (PMC3600027; doi:10.1186/1471-2164-14-165)
Supplement: Additional file 8 — Numbers and sizes of deletions and insertions supported by at least four reads in breast cancer genomes. [file 1471-2164-14-165-S8.pdf]

Additional file 8 - Numbers and sizes of deletions and insertions supported by at least four reads in breast cancer genomes.

Tumor sample	Deletion				Insertion			
	Total	Mean size (bp)	Variance (bp)	Median size (bp)	Total	Mean size (bp)	Variance (bp)	Median size (bp)
113T	141	820997	4880460	5598	50	1129	219	1161
114T	108	2012838	7712932	6085	17	1119	158	1105
116T	11	7366	7937	4897	4	979	268	1033
117T	8	18275664	39885834	8116	0	-	-	-
118T	10	1021748	2103098	5362	3	1327	49	1392
119T	7	5430	1124	5201	0	-	-	-
120T	7	507345	1224757	8457	837	636	179	624
147T	4	5356	1250	5173	0	-	-	-
148T	16	536405	2057946	4742	47	1064	446	1038
149T	38	558983	1759330	4960	27	1345	342	1492
150T	12	12699	27199	4780	638	624	223	599
151T	6	3087943	6893014	5832	3	1194	273	1565
152T	3	4737	280	4976	2	1434	85	1519
153T	30	6415	5635	4576	48	639	292	610
154T	18	5162	1491	4795	13	1507	131	1588
