# Supplementary material for: Gene rearrangements in hormone receptor negative breast cancers revealed by mate pair sequencing
Source: BMC Genomics. 2013 Mar 12;14:165. doi: 10.1186/1471-2164-14-165 (PMC3600027; doi:10.1186/1471-2164-14-165)

Additional file 9 – Flowchart of filtering structural variants (SV) identified in 15 tumor samples. See Additional file 2 for numbers of SVs in each sample before and after filtering.

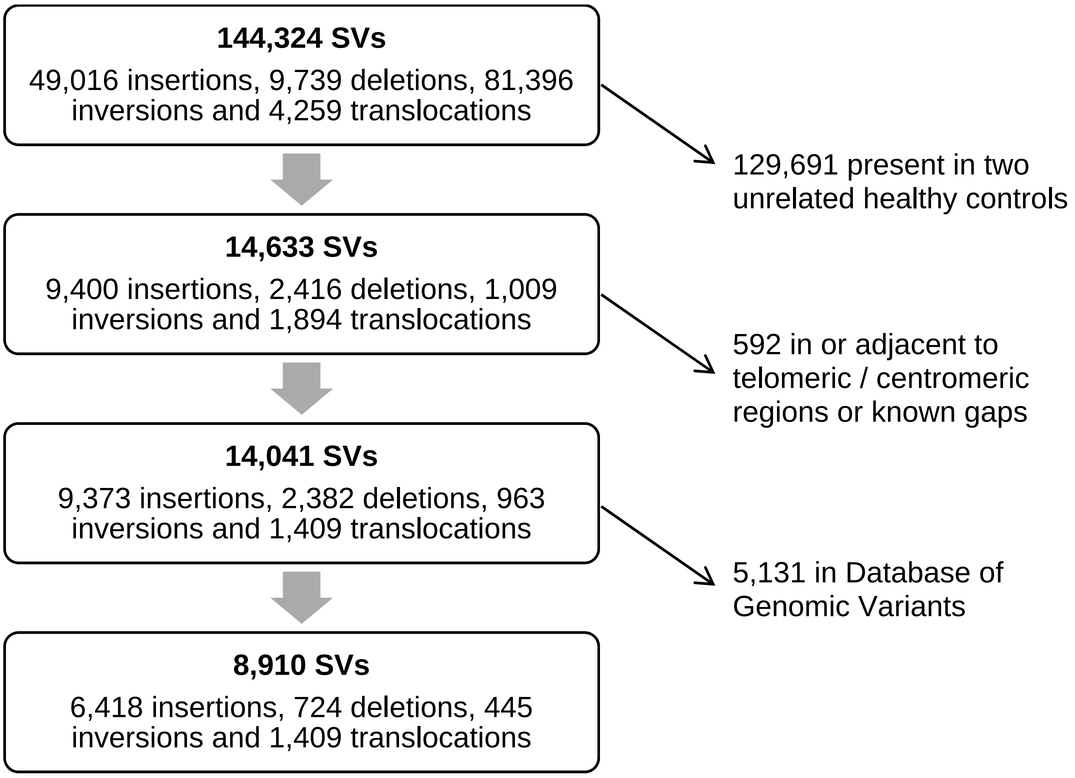

Supplement: Additional file 9 — Flowchart of structural variants (SV) identification procedures. [file 1471-2164-14-165-S9.pdf]
